# P-IOTA: A Cloud-Based Geographically Distributed Threat Alert System That Leverages P4 and IOTA

**DOI:** 10.3390/s23062955

**Published:** 2023-03-08

**Authors:** Amir Al Sadi, Carlo Mazzocca, Andrea Melis, Rebecca Montanari, Marco Prandini, Nicolò Romandini

**Affiliations:** Department of Computer Science and Engineering, University of Bologna, 40136 Bologna, Italy; amir.alsadi@unibo.it (A.A.S.); carlo.mazzocca@unibo.it (C.M.); a.melis@unibo.it (A.M.); rebecca.montanari@unibo.it (R.M.); nicolo.romandini@unibo.it (N.R.)

**Keywords:** SDN, P4, IOTA, Tangle, blockchain, distributed ledger technology

## Abstract

The recent widespread novel network technologies for programming data planes are remarkably enhancing the customization of data packet processing. In this direction, the Programming Protocol-independent Packet Processors (P4) is envisioned as a disruptive technology, capable of configuring network devices in a highly customizable way. P4 enables network devices to adapt their behaviors to mitigate malicious attacks (e.g., denial of service). Distributed ledger technologies (DLTs), such as blockchain, allow secure reporting alerts on malicious actions detected across different areas. However, the blockchain suffers from major scalability concerns due to the consensus protocols needed to agree on a global state of the network. To overcome these limitations, new solutions have recently emerged. IOTA is a next-generation distributed ledger engineered to tackle the scalability limits while still providing the same security capabilities such as immutability, traceability, and transparency. This article proposes an architecture that integrates a P4-based data plane software-defined network (SDN) and an IOTA layer employed to notify about networking attacks. Specifically, we propose a fast, secure, and energy-efficient DLT-enabled architecture that combines the IOTA data structure, named Tangle, with the SDN layer to detect and notify about network threats.

## 1. Introduction

The advent of distributed technologies has led to the emergence of decentralized systems that rely on a network of nodes for computation and data storage. These systems facilitate the collaborative and distributed use of computational resources, as opposed to relying on a central authority, leading to more efficient resource utilization, improved security, and greater resilience. However, the very nature of distributed infrastructures intrinsically brings architectural vulnerabilities that can be exploited by attackers. One of the most-famous attacks that leveraged these architectures is the distributed denial of service (DDoS) [1], which seeks to disrupt network services and host connectivity in a distributed environment by overloading the network with unnecessary requests. Avoiding and mitigating DDoS attacks is a primary concern for many organizations.

Software-defined networking (SDN) is a cutting-edge networking approach that divides the control and data plane layers to carry out its specific tasks. In SDN, the physical network layer is seen as fully programmable, resulting in increased customization of data packet processing. Such a feature has greatly contributed to its widespread deployment across different cloud infrastructures. In this direction, the Programming Protocol-independent Packet Processors (P4) has emerged as an innovative programming language, operating at the data plane level, to configure network devices in a highly customizable manner. P4 allows full programming of networking devices while being target-independent. Furthermore, the capability of programming the data plane is boosting the ability to detect network attacks.

How to effectively and securely share information to detect attacks is a challenging task. Distributed ledger technologies (DLTs), such as blockchain, are digital systems spread across multiple locations that securely store information (transactions) without the need for a central entity. Blockchain represents the most-famous type of DLT; its data structure foresees a chain of blocks connected through hashes and validated by third-party entities (i.e., miners or validators) following a consensus protocol. However, the validation process consumes a significant amount of time and energy, which can hinder blockchain’s efficiency and adoption for information sharing. To address these concerns, alternative DLTs, such as IOTA, are recently emerging as promising solutions. IOTA offers the same security features of the blockchain (i.e., immutability, traceability, and transparency) while addressing the efficiency concerns.

The differences between IOTA and blockchains have been widely investigated in the literature [2,3,4], with numerous studies showing that IOTA is a superior solution in terms of scalability, transaction rate, and efficiency, particularly with regard to energy consumption. IOTA outperforms traditional blockchains due to its low latency combined with the ability to send transactions without any fees [2]. Reference [4] presented an in-depth comparison of the performance of multiple consensus protocols where IOTA achieves the best performance with a transaction rate that is several orders of magnitude higher than the other protocols. Therefore, its lightweight consensus protocol makes it one of the few truly suitable technologies for Internet of Things (IoT) devices [3]. These properties are particularly relevant in SDN-based environments due to the strict latency requirements of the data plane. In the state-of-the-art, there are very few examples of research proposals that integrate P4 and DLTs. We argue that one of the main reasons is that traditional blockchain-like technology, such as Ethereum, introduces considerable overhead to compute blocks, clashing with the real-time attack detection capabilities of P4.

This paper presents P-IOTA, a system that leverages P4 and IOTA to detect ongoing network attacks in real-time. Our solution offers high performance and enables the real-time communication of events in a distributed manner. P-IOTA can be employed to address a wide range of attack scenarios. We developed a proof-of-concept using a well-known P4 implementation for DDoS attack detection, and we simulated an SYN flooding attack. The experimental results demonstrated that P-IOTA outperformed blockchain-based proposals in detecting and communicating attacks in real-time, as well as correcting false attack notifications that may occur.

The paper is organized as follows. In Section 2, we introduce the relevant background information regarding SDN, P4, and DTLs. The relevant literature is reviewed in Section 3 to present the background concepts used in our work and to highlight the limitations of existing solutions. In Section 4, we describe P-IOTA, including its main components and features. Then, Section 5 documents the experimental results of a simulated SYN flooding attack. Finally, Section 6 draws our conclusions.

## 2. Technical Background

In this section, we provide essential background information about SDN, the P4 programming language and its basic constructs, and DLTs with a specific emphasis on IOTA.

### 2.1. Software-Defined Networking

**Figure 1:**
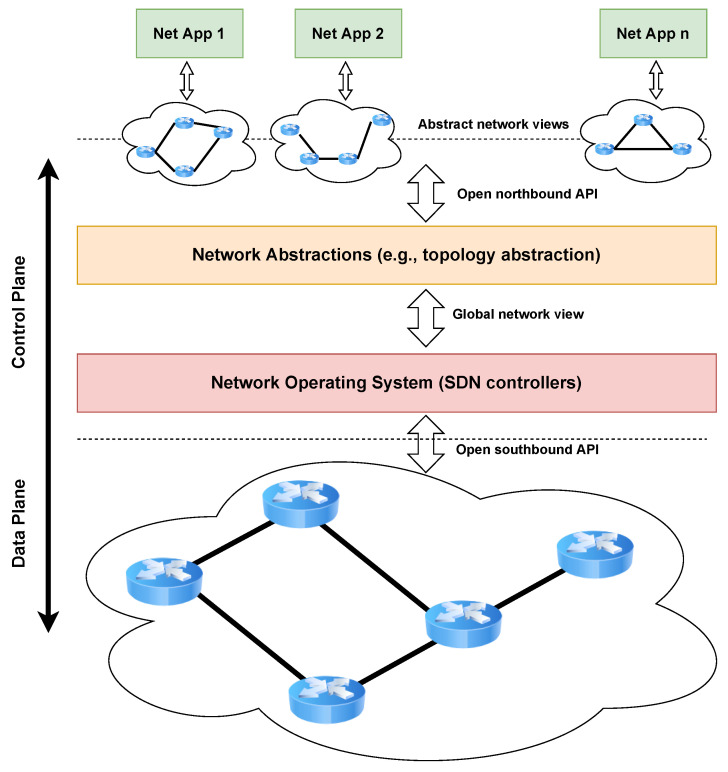
The SDN architecture.

Traditional network configurations are performed by individual design and installation of forwarding rules on packet-handling devices, hindering the ability to deploy configuration updates dynamically and cohesively. To solve this problem, the Open Networking Foundation https://opennetworking.org (accessed on 20 December 2022) promoted SDN, a highly dynamic, cost-effective, and easily adaptable network architecture. The basic idea behind this paradigm is to decouple the control plane, which selects forwarding behavior policies, from the data plane, which is responsible for forwarding packets. Figure 1 shows the layers of SDN architectures. The data plane is composed of the SDN-enabled switches, which use a flow table to encode the forwarding rules. The control plane comprises one or more physical or virtual devices, known as controllers, that fill the flow table of the SDN switches. The layers communicate using APIs: the Northbound Interface API is the channel between the control plane and the applications, while the Southbound Interface API connects the control and data planes.

The SDN paradigm brings numerous advantages, such as the ability to directly program all components of the underlying network, abstracting the control plane from the forwarding plane, allowing operators to directly adjust traffic flow according to the user needs, to increase reliability due to centralized network control, to implement new services without the need to configure individual devices, and to improve network automation and management. Thus, this type of infrastructure requires the data plane devices to communicate with the control layer devices via some type of protocol: an example is the OpenFlow protocol, a standard communication interface defined between the controller and the SDN switch.

#### SDN Attacks

Throughout SDN’s lifespan, the scientific literature has pinpointed a wide variety of vulnerabilities and proposed workarounds for them. As the literature suggests [5], vulnerabilities can be grouped according to the SDN layers. In this section, we chose to describe some threats that can affect the data and control plane. Side-channel attacks are the first type of attacks we considered. In this scenario, the intruder analyzes the time gap or the flow configuration delay in the flow table to fetch the network configuration. This attack is possible due to the lack of confidentiality on the data plane. Fingerprinting is usually employed to counteract these threats [6,7].

On the other hand, man in the middle (MiTM) attacks affect the control planes: in this scenario, attackers are able to sniff traffic that flows from a sender to a receiver. This attack can be easily enforced in the Southbound Interface, due to the fact that the channel is usually not encrypted. Adhikari et al. [8] proposed an MiTM mitigation scheme that leverages ECDH and AES encryption to encrypt information sent between the data and control plane.

However, one of the most-disruptive data-plane-based attacks is denial of service, an attack that aims at making a machine or network resource unavailable to its intended users by disrupting the services of a host connected to a network. The scientific literature has focused in depth on finding ways to mitigate these sorts of attacks. Fouladi et al. [9], for example, proposed a scheme to counteract DoS attacks by employing an auto-encoded neural network to mitigate a set of different types of DoS attacks.

In our work, we focused on data-plane-based attacks such as DoS, since we are able to mitigate traditional SDN vulnerabilities by employing the programmable data plane.

### 2.2. P4

P4 [10] is an open-source programming language designed to control packet processing in the data plane. A P4-enabled switch provides two innovative concepts: (i) the switch’s functions are defined by the P4 program, rather than predetermined; (ii) the communication between the control and data plane occurs in a fixed-function device channel, but the data plane APIs are defined by the P4 program. P4Runtime [11] offers the specifications for abstracting the hardware interfaces and building the Southbound Interface APIs that expose the specific features and protocols supported by the data plane [12]. The main goals of P4 are the following:

- Reconfigurability: The controller can dynamically install and update the packet parsing logic and processing rules.
- Protocol independence: The controller can specify how to process header fields providing rule names, key types, and typed match + action tables, breaking the tie that standard switches have to fixed packet formats.
- Target independence: The controller programmer can design behaviors independently of the details of the underlying switch. The task of translating program features exploiting target-specific capabilities is delegated to the P4 compiler.

P4 is built around an abstract model that describes the switch’s traffic forwarding process through multiple stages of match+action, arranged in series, parallel, or a combination of both. Inbound packets are first handled by the parser, which extracts header fields and acts as a programmable interpreter of supported protocols. The extracted header fields are then passed to the match+action tables, which determine the egress port and queue for the packet. Based on the ingress processing, the packet may be forwarded, replicated, dropped, or trigger flow control. A P4 program expresses the behavior of the data plane, defining the following components:

1. Header types: packet header definitions, i.e., the set of fields and their sizes.
2. Parsers: finite-state machines that map packets into headers and metadata.
3. Tables: data structures defining matching fields and actions applied to them.
4. Actions: code fragments that describe packet manipulation and can consider external data, supplied by the control plane at runtime.
5. Match–action units: elements that construct lookup keys from packet fields’ metadata and use them to find the right action and execute it.
6. Control flows: imperative blocks that describe packet processing on a target using the data-dependent sequence of match–action unit invocations.

P4’s unmatched expressiveness gave a disruptive new perspective on network programmability and monitoring while increasing the scientific community’s interest in this topic. On the other hand, it does have entry barriers, such as the need for enabled hardware and the effort required for network architects to become proficient in designing efficient and portable code.

### 2.3. Distributed Ledger Technology

Distributed ledger technologies (DLTs) are a type of distributed database that avoids the centralization of data and does not require administration functionality. The stored information is replicated on multiple nodes that maintain a copy of the entire database. Since there is no centralization or third-party entities, the data source is built collaboratively, allowing multiple entities to contribute data. Unlike traditional databases, data memorized on a DLT can neither be modified nor deleted, as they are usually implemented as append-only data structures. They rely on peer-to-peer (P2P) networks as they are decentralized systems. The lack of a centralized control entity avoids the single point of failure issue. For this reason, DLTs adopt consensus protocols to keep the nodes in the network synchronized. Trust between participants is established through these protocols, which are based on strong cryptographic principles.

It is possible to distinguish many categories of DLTs according to specific characteristics. The first factor is the data structure chosen to store the information. The most-popular are blockchains and directed acyclic graphs (DAGs). A blockchain, as the name suggests, stores information in blocks linked together by hash pointers. This makes it possible to notice tampering with data since changing a block would break the chain. A directed acyclic graph is a data structure no longer organized as a linked list of blocks, but as a directed graph without cycles in it. DLTs can be divided into two different access models: permissionless and permissioned. In the first model, the ledger is public and open access; hence, anyone can participate in the network and the consensus protocol. It is fully decentralized across unknown parties. In the second model, participation is mediated by permissions: participants have restrictions on writing or both reading and writing. In general, it is partially decentralized. DLTs can also be classified into tokenized and tokenless ledgers. In a tokenized ledger, transactions involve some type of purely digital asset (token) represented within the ledger. Tokens generally serve two main purposes. The first is that they act as an economic incentive for protocol participants to form consensus in decentralized systems. Forming economic incentives within a consensus protocol is only relevant for decentralized systems (a miner mining a block outside the consensus wastes energy and forgoes any reward). The second purpose is that they help prevent spam and DoS attacks. In fact, each operation involves a nominal fee to be performed. Spammers can be hampered by the enormous costs involved in creating a large number of transactions. In a tokenless scenario, the ledger does not offer any good as an incentive to join or expect any payment for implementing smart contracts. For this reason, tokenless ledgers are typically permissioned, and thus, a strong trust has already been established during the registration process. Some ledgers also allow the simulation of a Turing machine. For example, Ethereum or Hyperledger Fabric can execute Turing machines. This allows programs written in Turing-complete programming languages to be stored and executed directly on the ledger. These programs are often called smart contracts.

### 2.4. IOTA

**Figure 2:**
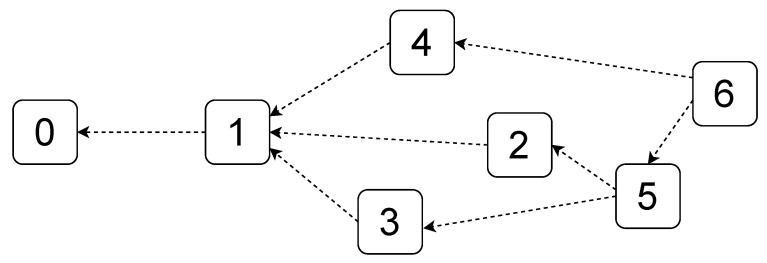
The Tangle.

First-generation blockchains exhibit significant efficiency issues [13] that make them unsuitable for environments where resources can be extremely heterogeneous (i.e., IoT). IOTA [14] is a next-generation DLT engineered to tackle the scalability limits of the blockchain while still providing the same security capabilities such as immutability, traceability, and transparency. IOTA owes its high scalability to the adopted data structure named Tangle (sketched in Figure 2), a DAG composed of several connected nodes that store transactions. Each node is a transaction, while each edge represents a validation of that transaction. The Tangle enables achieving remarkable performance due to the lack of a middleman since there are no block producers (i.e., miners and validators). Thus, everyone can submit a transaction and attach them to different nodes. However, in order to achieve a secure shared state, a new transaction has to verify the two transactions to which it is directly connected. Furthermore, since transactions are not validated by someone that has to be rewarded, it also enables zero-value transactions. This feature is particularly relevant for certain scenarios where there are a huge amount of data to send, resulting in an extremely large number of transactions. Since zero-value transactions do not involve any transfer value, they are attached to the Tangle without the need to be validated by participants of the network (i.e., double spending cannot occur), thus remarkably reducing the time to share information.

An IOTA network can be deployed as private or public. A private network only provides access to certain users. On the other hand, a public network can be accessed by anyone without any kind of restrictions: every participant is aware of the history of transactions and sent new transactions.

IOTA distinguishes clients and nodes. A client is any entity (i.e., human or not) that submits transactions to a node, to have them attached to the Tangle; nodes have to verify the correctness of the transactions and, in the case of success, add them to the Tangle. Furthermore, an IOTA network comprises additional node types named Coordinator and Permanode. In each IOTA network, there is a unique Coordinator that regularly produces milestones, trusted signed transactions used by nodes to confirm transactions. The signature guarantees that nobody can fake the signatures on milestones; thus, milestones are always legitimate. In particular, a transaction is confirmed only when directly or indirectly referenced by a milestone that nodes have validated. The use of the Coordinator is temporary; it will soon be removed in incoming updates. Permanodes are responsible for keeping the history of all the transactions that occurred. Such a component is particularly relevant in specific scenarios since nodes may be constrained devices that cannot memorize the entire Tangle. Therefore, they periodically delete recorded transactions using a pruning operation.

## 3. Related Work

This section reviews existing works on the integration of blockchain with SDN and P4. Furthermore, it also analyzes some research efforts that employ P4 for thwarting SYN flooding attacks.

### 3.1. SDN and Blockchain

The combination of SDN and blockchain can find various applications, motivated by both the necessity to address SDN’s inherent security issues and the opportunities to manage the energy consumption of devices [15]. By exploiting the distributed architecture that blockchains are based on, some researchers combined those technologies and applied them to IoT infrastructures to pursue various intents. Yazdinejad et al. [16] proposed a specific architecture to provide an efficient and secure mechanism for file transferring between IoT devices, to overcome the computational limitations of such devices. IoT devices are clustered around their respective SDN controller and are able to communicate over a P2P network using a public blockchain. The computational need is eased by removing the proof of work (PoW) process thanks to the controller’s role, the clustered nature of the architecture, and an ad hoc distributed trust algorithm. Inside the clusters, a private blockchain is used to keep track of the newly added IoT devices and every transaction. To transfer a file between devices, a preliminary block that contains the sender and receiver signature and public keys is designed. After the block is validated by the network, the file is then sent to the intended recipient, which is the only one that can decode it. A similar use case was shown in [17], where with the use of OpenStack and Pythereum, a blockchain-enabled SDN was implemented and tested. The role of the blockchain in this architecture is to present indelible and transparent records of any file transfer, which the network then validates. Jiasi et al. [18] presented a proof-of-concept practical design in which a blockchain layer was placed between the control and data layers, to record network events and resources associated with every controller and build smart contracts that automatically implement security protocols.

To tackle the single point of failure (SPOF) architectural vulnerability, while having the purpose of enhancing SDN’s security level, Abou et al. [19] proposed an architecture that incorporates the blockchain as a way to make multiple SDN-based domains collaborate and share DDoS attack information in a decentralized manner. This work exploited a smart contract where collaborators can publish real-time IP addresses of malicious parties that need to be blacklisted. The authors deployed the smart contract on an Ethereum testbed network to evaluate the infrastructure, which resulted in being flexible, secure, and low-cost. The choice of deploying the solution on a public blockchain enabled information sharing between different clouds to achieve collaboration, especially needed in IoT environments, as shown in [20].

Rahman et al. [21] presented a framework that exploits the Ethereum blockchain to publish all the flow rules of the switches: the controller periodically creates a block as an update only if all the switches agree on the proposed list of rules. The immutability and consistency of the blockchain allow the management of flow rules and the detection of their violations on devices. However, the authors reached the conclusion that deploying this kind of architecture in the real world is rather complex because of the amount of transactions needed, which can entail a considerable cost. Similarly, Sharma et al. [22] presented a distributed secure SDN architecture for the IoT using the blockchain technology concept to improve security, scalability, and flexibility, without the need for a central controller. The blockchain was employed as a distributed peer-to-peer network where non-authenticated members can interact with each other without a trusted intermediary. The blockchain is deployed in order to allow this untrusted interaction to update a flow rule table, securely verify and validate a version of the flow rule table, and download the latest flow rules table for IoT forwarding devices. In addition, the DistBlockNet architecture provides proactive and reactive incident prevention by dynamically adapting to the threat landscape without having to include security administrators to manually process a huge number of advisories and approvals.

### 3.2. Blockchain Interaction with P4-Enabled Switches

The efficiency of the solutions described in Section 3.1 has been validated by comparing it with existing models, as highlighted in [23]. However, we believe they did not fully exploit the potential of the programmable data plane. To the best of our knowledge, only two solutions attempted to integrate blockchain and SDN by leveraging the P4 language.

Febro et al. [24] presented a botnet DDoS defense framework using P4, SDN, and blockchain at the network edge. It implements a synchronized defense within an organization or also spanning multiple organizations. The framework comprises two main agents, ShieldSDN and ShieldCHAIN. ShieldSDN is an SDN controller managing edge devices with P4 capabilities, responsible for synchronizing packet filters provided by the switches within an organization. ShieldCHAIN is a distributed application that leverages a smart contract deployed in the Ethereum blockchain. ShieldCHAIN is responsible for inter-organization synchronization: when a publisher or organization wants to share attack fingerprints with the community, ShieldCHAIN creates a transaction. The subscriber can then retrieve the current state of attack, and ShieldCHAIN then installs the required countermeasures in the data plane. The authors performed four experiments to validate their solution, i.e., to prove that ShieldCHAIN is effective at orchestrating the programmable data plane, control plane, and blockchain in a scalable solution against botnet-based DDoS, by synchronizing packet filters on the edge networks. Acknowledged drawbacks included the high request of computational resources to run the public blockchain and its intrinsic delay.

Yazdine et al. [25] proposed a P4-based blockchain-enabled packet parser (BPP) located in the data plane and implemented on an FPGA. The BPP implements a custom header in P4 that fits the blockchain structure. The BPP is able to recognize blockchain hash blocks to enforce control policies, such as match+action tables using specific fields in these packets. The workflow outlined by the authors involves the data plane, the control plane, and the application layer. It begins with packet processing in the BPP module, which leverages the intrusion detection functions in relation to attack types (five categories of patterns: normal, remote to local (R2L), DoS, user to root (U2R), and probing attack (probe)) to detect attacks. If an anomaly is detected, a transaction is prepared to be validated and then added to a block in the ledger for that specific attack. Then, the BPP submits a transaction to a validator in the control plane and notifies the controller of that attack detection. Subsequently, the SDN controller can use the Merkle tree to evaluate the transaction. If this process is successful, the transaction will be marked as valid in the whole network. Concurrently, the blockchain will be updated and the BPP will be re-programmed, if necessary.

### 3.3. P4 for Thwarting SYN Flooding Attacks

SYN flooding attacks are a type of attack that attempts to concurrently establish a large number of connections to disrupt the networking capabilities. The attack involves flooding the network with SYN/ACK TCP packets targeting a host in an effort to establish TCP connections and block the available ports. P4 is a promising candidate for designing and deploying in-network detection and mitigation strategies for SYN flooding attacks. It allows keeping track, in real-time, of the ratio of SYN/ACK sent in a flow compared with the corresponding ACK/FINs. The literature shows how real-time data plane detection enables accurate assessment of whether a network flow is malicious or not. One such implementation was reported in P-SCOR [26], where a simple asymmetric-flow detection algorithm was proposed.

Shen et al. [27] proposed a P4-based SYN and UDP flooding mitigation strategy that combines the two steps of attack identification:

1. Source authentication: by using an SYN cookie, the source of traffic is authenticated in the system.
2. Anomaly detection: after the authentication step, the real three-way handshake takes place and the P4 program supervises the correctness of the process.

**Figure 3:**
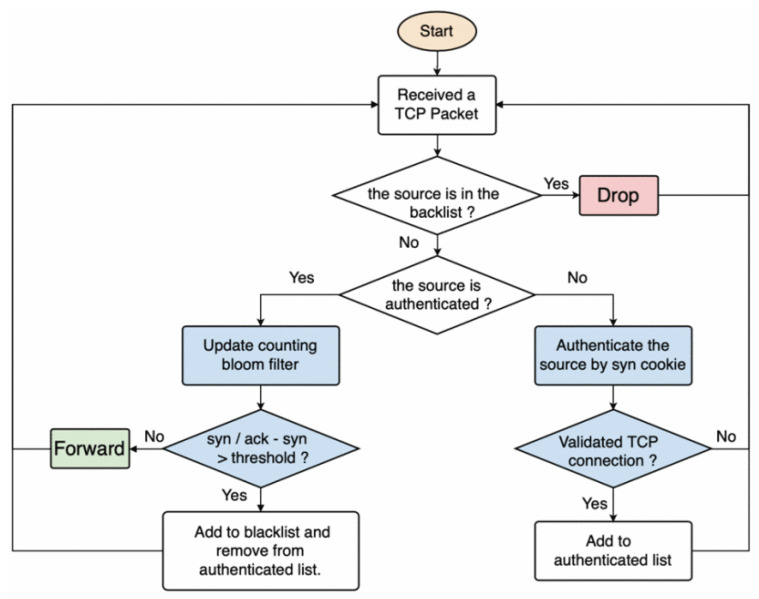
Two-step P4-based SYN and UDP flooding mitigation and identification strategy.

Such operations, whose flow is shown in Figure 3, allow early detection of ongoing attacks on the authentication phase and prune the remaining possible threatening flows in the second phase. The results show a drastic reduction in the server SYN queue usage when the P4 firmware is deployed in the network. Similarly, another three-step solution was proposed by Lin et al. [28]. Different from [27], the authors merged overlapping switch rules to minimize the number of dropped benign flows. Three concurrent components make up the solution. A detection oversees the ratio of SYN/ACK and ACK/FIN packets related to each network flow. Meanwhile, a merging phase is employed: here, multiple entries on a switch are simplified to a larger prefix to minimize the number of installed rules. The defense mechanism matches each attacker in a flow table rule: if the rule installed in the switches is LPM and an attacker is in that IP range, the rule is deleted, while if the rule is an exact match, the IP is dropped.

SYN flooding attacks are one of the most-threatening attacks in distributed environments; hence, we claim they can be a remarkable example to showcase the potential of our solution.

The scientific research on P4 solutions that incorporate blockchain technologies is limited. Most of the existing solutions choose the Ethereum blockchain, and the focus is mainly on security applications in IoT environments. There is a lack of studies on P4 and blockchain in cloud environments and few works that leverage the blockchain to propagate P4 alerts in the case of detected attacks. To the best of our knowledge, there are no works that employ alternative DLTs such as IOTA, which has been shown to be more efficient than popular blockchain-based solutions, especially for IoT environments.

### 3.4. Considerations about P4 Employment in Blockchain Solutions

The scientific literature on P4 solutions that exploit blockchain technologies is very limited, with most of the existing solutions choosing the Ethereum blockchain. Table 1 shows what are the main topics treated by each paper mentioned in the related works. These works mainly focus on security applications in IoT environments. Two of the analyzed works focused on recording events, while only one covered file transfers. By reviewing the literature, we observed a lack of studies on P4 and blockchain in cloud environments and few works that leverage the blockchain to propagate P4 alerts in the case of detected attacks. On the other hand (as shown in Section 3.3), a large number of works use P4 as the enabling technology to deploy detection strategies to spot abnormal behaviors on the data plane, i.e., SYN flooding attacks.

To the best of our knowledge, there are no works that employ alternative DLTs such as IOTA, which has been shown to be more efficient than popular blockchain-based solutions, especially for IoT environments.

## 4. P-IOTA Architecture

**Figure 4:**
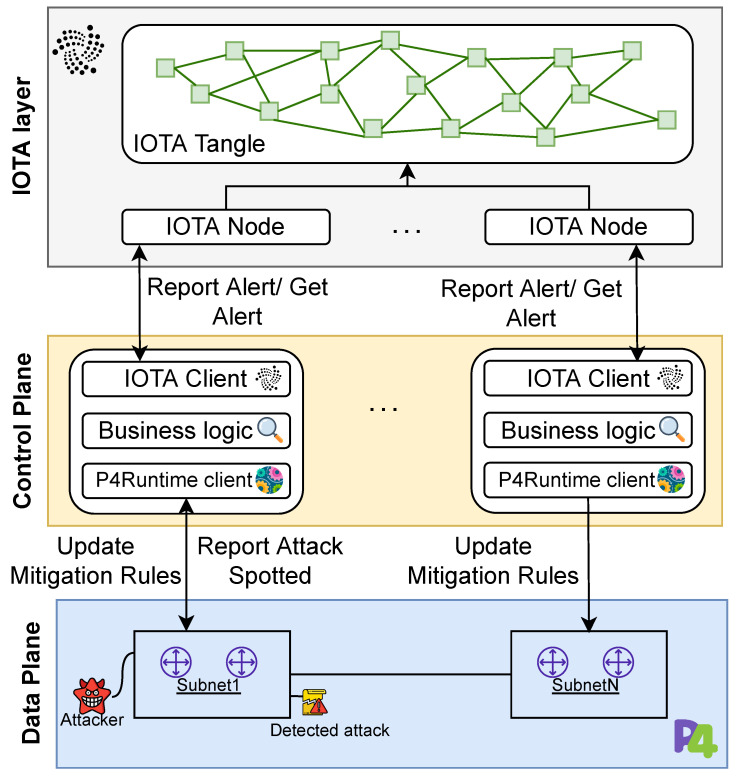
The P-IOTA architecture.

In this section, we present the design of our solution, which is depicted in Figure 4.

P-IOTA is tailored for distributed networks that belong to multiple organizations, such as cloud infrastructures, where computational and networking resources are often spread across geographic locations and critical information is stored. These types of networks are often treated as local networks (e.g., cloud-hosted installations) by system administrators, but it can be challenging to quickly detect and block threats while spreading alert information in such a distributed environment.

The main goal of this framework is to facilitate the dissemination of network attack alerts through IOTA. In order to generate alerts in a highly customized and efficient way, P-IOTA leverages an SDN-based architecture, involving a P4 data plane layer that detects network attacks. The generated alerts are then used to notify other controllers through the IOTA layer. The infrastructure consists of three main components:

- IOTA layer’s main role is to notify and log alarms from the data plane and to share mitigation strategies. The IOTA layer notifies the portions of the network that can be impacted by the detected attack and disseminates the policy that should be applied to mitigate it.
- The control plane is responsible for managing and configuring the underlying physical network. It contains multiple local network managers (i.e., controllers), each of which controls a specific subnet. The controllers interact with the IOTA layer using an IOTA client and with the data plane using P4Runtime.
- The data plane is the layer hosting the physical devices that forward traffic. By using P4 and the programmable data plane, part of the detection intelligence can be moved from the control plane to the data plane. This allows for deep packet inspection and network-level probing to detect anomalies.

P-IOTA is designed to propagate real-time alerts from the data plane and deliver them quickly throughout a distributed environment. The IOTA Tangle maintains an immutable list of alerts in the form of a log, allowing for further investigations into the attack history offline without the risk of log cleaning. Moreover, the Tangle can be leveraged to share the countermeasure needed to mitigate the detected attacks. Hence, our solution offers intrusion detection capabilities on the data plane in the form of in-network detection, offloading a significant amount of detection intelligence to networking devices.

In this paper, we demonstrate how IOTA significantly reduces the overhead compared to traditional blockchain solutions, highlighting its potential in the network security field.

### 4.1. IOTA Layer

The IOTA layer comprises the IOTA nodes that hold a unified view of the Tangle. Our decision to use IOTA to share information across different sites [29] was motivated by the following features:

- Efficient lookup: Each transaction can be tagged, making it easier to collect IPs from the Tangle. If another DLT were adopted, an additional tag within the transaction message would significantly slow down the time it takes to find a transaction.
- Zero-value transactions: IOTA enables neglecting cryptocurrency, reducing the complexity of managing IPs. In contrast, each controller would need to have sufficient funds to perform the operations.
- Scalability: The Tangle allows parallel validation of transactions without any intermediary. Such a capability overcomes blockchain-based solutions, where a transaction is not recorded until it is stored in a block.

Finally, the Tangle structure also shortens the time needed to record a new transaction. Transactions are recorded on the Tangle as soon as they are created, whereas, in blockchain-based solutions, they must wait until they are stored in a block.

#### 4.1.1. IOTA Node

In a federation, each participating enterprise should have at least one IOTA node to receive notifications from other organizations. However, a company may not collaborate with external parties and may have multiple sites located in different regions. Therefore, to reduce latency and facilitate swift mitigation, a company may choose to deploy an IOTA node at each of its sites.

#### 4.1.2. IOTA Tangle

The Tangle is the data structure employed to share information, such as alarms and mitigation, among different controllers. This information is shared through zero-value transactions that do not require validation and, hence, help maintain a unified view of the Tangle while keeping low latency and energy consumption. As discussed in the previous section, these features make IOTA a suitable choice for SDN-based scenarios where threat alerts and mitigation have to be quickly disseminated among devices that may have limited capabilities.

### 4.2. Control Plane

**Figure 5:**
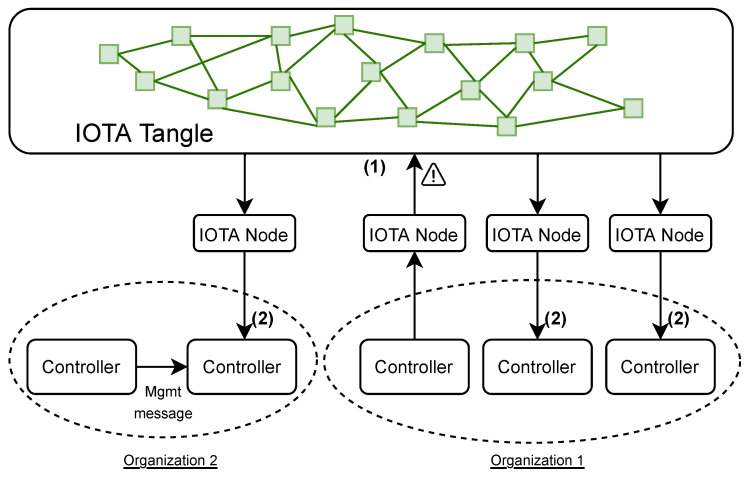
An example of organizations managing controllers associated with different subnets. In a federated environment, if an alert (1) is generated from a node, all interested controllers across organizations are notified (2).

The control plane is responsible for managing, configuring, and monitoring the physical network. It consists of multiple geographically dispersed controllers, which are in charge of managing a single network. These controllers work together and receive alerts from the IOTA layer, which informs the correct nodes of potential attacks, as shown in Figure 5.

Each controller acts as the primary management point for a local network. It keeps track of the status of networking devices, communicates with other controllers to make decisions about local network management strategies, and provides common control place services to monitor and administrate the data plane.

The control plane is made up of multiple controller instances, which are connected through messages. The components of each controller are:

- The IOTA client is responsible for connecting the controller instance to the corresponding IOTA node in the IOTA layer. It communicates with the IOTA node to send and receive alerts.
- The controller business logic handles the forwarding of the alerts to the IOTA client, sending management messages (e.g., congestion, link failures, etc.) to other controllers, and communicating with P4Runtime.
- P4Runtime is in charge of interacting with the networking through the P4Runtime Southbound Interface. It receives alerts generated from the data plane and installs the rules to react to these alerts.

As highlighted in Figure 5, organizations may manage multiple controllers, and if a controller detects a potential attack, it will communicate it to the IOTA node, which then notifies the interested controllers through the IOTA Tangle.

#### 4.2.1. IOTA Client

The role of each controller in detecting and mitigating attacks is accomplished through the integration of an IOTA client. This client serves as a bridge between the controller and the IOTA node, allowing the exchange of information between the controller and the IOTA Tangle. One of the advantages of using IOTA clients is their lightweight design, which makes them suitable for deployment on devices with limited resources.

#### 4.2.2. Controller Business Logic

This component plays a key role in managing and controlling the underlying network. It is responsible for expressing policies and communicating configuration or resource changes to neighboring controllers via management messages. The management messages are used to exchange information between physically close controllers, while the IOTA layer is in charge of disseminating alarms and mitigation strategies across a distributed network. In summary, the main functions of a controller include:

- Management messaging: sending messages to communicate with the neighbors’ controllers.
- Alerting: forwarding alerts coming from the data plane to the IOTA node for further dissemination.
- Network configuration: interacting with and configuring the underlying network for forwarding or mitigation purposes.

This controller acts as the centralized core that manages the subnet and demonstrates the intelligence of the administration.

#### 4.2.3. P4Runtime Client

This component is the client for the Southbound Interface that connects the controller business logic and the data plane level. As outlined in Section 2.2, P4Runtime abstracts the underlying hardware or software and offers agnostic APIs to the control plane to communicate with the physical network. The P4Runtime client is responsible for receiving communications from the data plane and performing two key functions:

- Installing match-action rules: it installs rules that specify forwarding logic or threat detection and mitigation strategies.
- Event listening: it listens for alerts from the data plane that indicate potential threats.

### 4.3. Data Plane

**Figure 6:**
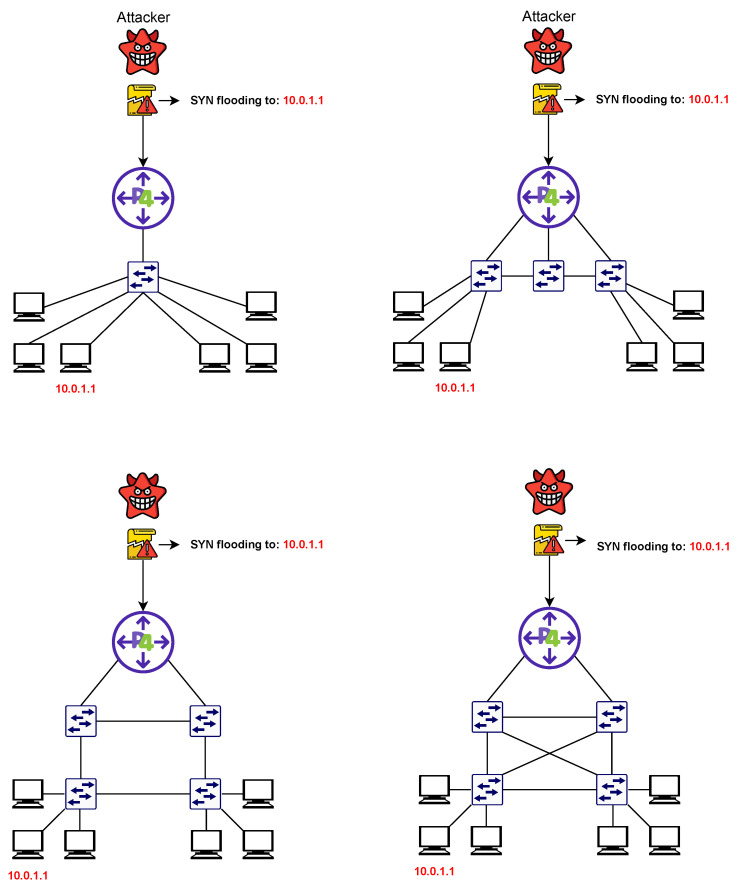
Network topology used for the testing phase. From the top-left corner, clockwise: single switch topology; linear topology; ring topology; fully connected topology.

The data plane is in charge of processing and forwarding traffic. It includes networking devices such as switches and routers. Each controller is paired with one or more P4 border routers, which are capable of monitoring the traffic flowing in a given subnet and detecting abnormal behaviors. This allows the P4 switch to centrally inspect each network flow and determine if an attack is taking place. The programmability of P4 and the data plane enabled us to design pipelines that incorporate the detection of ongoing network attacks and normal forwarding behaviors. In Figure 6, we report the network topology used for the experiments.

Over different possible attack scenarios, we focus on the two we considered more relevant:

- An organization is comprised of multiple physical subnets within the IOTA-controlled network. If an attacker is detected, the alert must be propagated to each geographically dispersed network subnet.
- The same physical network is used by multiple organizations (such as in public or hybrid cloud platforms). In this scenario, an attack may potentially affect each organization operating in that portion of the data center.

P-IOTA handles both of these scenarios in a consistent manner since each IOTA node is tied to its organization. Similarly, as depicted in Figure 6, each controller is connected to its IOTA node and can configure its network independently.

## 5. Case Study

To validate the proposed architecture and compare it with the existing literature, we considered a real-world use case scenario. This section aims to showcase a practical implementation of the SYN flooding detection and alerting workflow using the P-IOTA architecture. Therefore, we conducted a proof-of-concept evaluation of P-IOTA by focusing on SYN flooding, which is a common and harmful networking attack in distributed environments. This attack falls within the DDoS, notoriously known to disrupt the network forwarding capabilities and to leverage SDNs to threaten cloud infrastructures [30]. As discussed previously, the programmable data plane in P4 can mitigate these threats, as it addresses the centralized nature of traditional SDN controllers. There have been several successful implementations of P4 in mitigating DDoS attacks, demonstrating the effectiveness of the technology in securing distributed networks.

We conducted a proof-of-concept evaluation of our architecture by implementing an SYN flooding scenario. To detect the DDoS attack, we used the InDDoS solution proposed by Ding et al. [31]. This solution, which is fully located on the data plane, identifies potential DDoS victims based on data structures and thresholds. The solution has been validated with state-of-the-art datasets and has shown high detection precision. We deployed InDDoS using its open-source code available at https://github.com/DINGDAMU/INDDoS (accessed on 20 December 2022). We selected this solution as it aligns with our scenario: the Southbound Interface is minimally used, with each alert consisting of just 4 bytes (an IP). To simulate the network environment, we used Mininet [32] and Bmv2 [33] with a single switch and two host network topologies. The attack was generated using the Linux utility Hping3 [34].

### 5.1. Experimental Setup

We set up an IOTA network and evaluated the time it took to make all controllers aware of the victims’ IPs. To do this, we used zero-value transactions to share information on the IOTA network. The transactions were embedded with the attacked IP and were made immutable by the Tangle. However, this may lead to false positives if an IP is wrongly reported. Therefore, we enriched the message of transactions with an “action” field, which indicates the type of operation being performed (i.e., add or delete). To delete an IP incorrectly detected as suspicious, a controller has to send a transaction where the action field is set to “delete” and the IP field reports the wrong IP. An example of the message structure used to share information is shown in Listing 1.
**Listing 1.** Message structure.{     "action": <add | delete>,      "IP": <IP> }


IOTA enables binding a tag to a transaction, simplifying how IPs are collected. Each controller retrieves all the transactions indexed by a specific tag and builds the firewall rule table. In case multiple subnets are simultaneously attacked, IOTA will receive as many transactions as the number of detected attacks. All these transactions are indexed through the same tag. Hence, the controllers leverage that tag to retrieve all the corresponding alerts. The pseudocode of the algorithm implemented by IOTA clients is shown in Algorithm 1.
**Algorithm 1:** IOTA client.  **procedure** SendAlert      tag←“newAlert”      action,IP←getAlertFromController()      message←createMessage(action,IP)      sendToIOTA(tag,message)  **end procedure**  
**procedure** ReceiveAlert      tag←“newAlert”      messages←getMessagesFromIOTA(tag)  **for each**
m∈messages
**do**      action,IP←m      sendAlertToController(action,IP)     **end for**  **end procedure**


However, since the order of the collected transactions may be different from that of the detection, it is necessary to embed a temporal reference within each transaction, resulting in a different structure of the message shown in Figure 1. The controllers then use this information to reconstruct the temporal order properly.

In the scenario described, the primary concern is to detect and notify about an attack as soon as possible to minimize the attack window. Therefore, the following experiments were implemented:

- Experiment 1: notify about the detected attack—The first experiment aimed to evaluate the time needed to notify a controller from another organization about a detected attack.
- Experiment 2: update a wrong detection—The second experiment was about updating a wrongly reported alert.

Furthermore, to better justify the effectiveness of our solution, P-IOTA’s performances were compared to the framework presented in [24], which is the only work in the literature that employs a DLT for similar purposes. For the sake of fairness, we conducted the same experiments:

- Experiment 3: collect alerts—The third experiment measures the performances of the P4-based data plane layer.
- Experiment 4: publish—The fourth experiment refers to organizations that share information, such as the victim’s IP, with a community through transactions published on the Tangle.
- Experiment 5: subscribe—The fifth experiment involves community members that retrieve alerts previously published on the Tangle.
- Experiment 6: packet filter installation—The sixth experiment installs the appropriate filtering rules on switches based on the collected information to mitigate the ongoing attack.

### 5.2. Experiments

**Figure 7:**
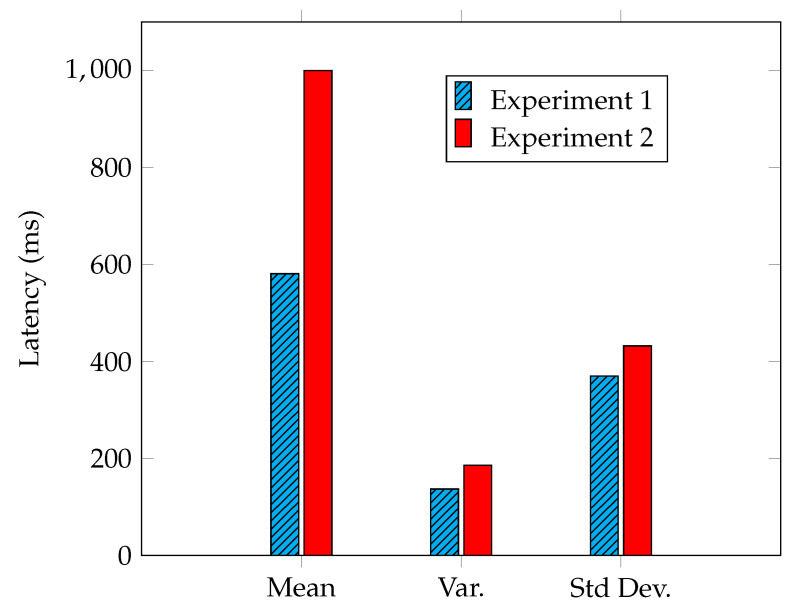
Experiment 1 and 2—Latency statistics.

**Figure 8:**
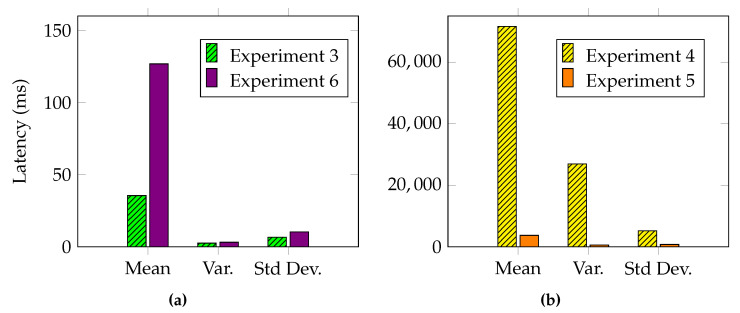
Experiments 3, 4, 5, and 6—Latency statistics for SDN components (**a**) and for the IOTA network (**b**).

Each experiment was conducted by simulating a workload of 100 detected alerts, which was repeated 100 times for accuracy and consistency. In the scenario under consideration, the main objective for a community is to synchronize a defense posture in the lowest possible time, so our analysis focused on the latency metrics required for the main operations. Figure 7 shows the results of the first and second experiments. In Figure 8, we devise the remaining experiments based on the components under evaluation. The results of the experiments that pertain to the SDN components can be seen in Figure 8a for Experiments 3 and 6, while the results of the experiments related to the IOTA network are shown in Figure 8b for Experiments 4 and 5.

### 5.3. Experiment 1: Notify about a Detected Attack

Firstly, we evaluated the time that elapsed between the notification of an attack by a controller and its availability to all the other controllers in the control plane. In particular, the elapsed time includes the creation of a transaction, its retrieval through indexing, and its conversion into a useful representation. The results are shown in Figure 7, where two types of latency time (declined among mean, variance, and standard deviation) are represented. We can claim that a notification, reported in blue, requires on average about 500 ms to make the alert available to other organizations.er.

### 5.4. Experiment 2: Update a Wrong Detection

In the second experiment, we evaluated the ability to update wrongly reported attacks. In this case, the average latency almost doubled. We expected such an outcome due to the immutability feature of the DLT. As a transaction cannot be removed from the Tangle, the modification involves two transactions: one to invalidate the previous one and another one to update it. The results are shown in red in Figure 7.

### 5.5. Experiment 3: Collect Alerts

The third experiment measures the time required by the P4 target to generate and send an alert to the controller. This time is the sum of the latencies collected in the following three steps:

- Create the digest packet describing the alert;
- Send it over the P4Runtime channel;
- Extract the alert in the control plane.

A programmable P4 switch allows for describing custom features that improve the performance of certain actions. This is reflected in the results of this experiment, as P-IOTA is able to shrink the content of the alert up to 4 bytes, i.e., the IP address of the victim. The comparison between P-IOTA and [24], whose results are reported in Table 2, demonstrated that P-IOTA outperformed the compared approach by three orders of magnitude. Figure 8a shows the mean, variance, and standard deviation of Experiment 3, collected over 100 measurements.

### 5.6. Experiment 4: Publish

The fourth experiment aimed to demonstrate the effectiveness of our proposal in sharing threat intelligence with the community. The results of the experiment, which involved embedding each detected alert within a transaction, are shown in Figure 8b. As the number of detected alerts increased, so did the number of transactions published on the Tangle. Thus, detecting 100 alerts would result in 100 transactions published on the Tangle. Our proposal performed better due to the fact that IOTA does not have the concept of blocks, allowing transactions to be attached to the Tangle as soon as they are collected by the underlying layers.

### 5.7. Experiment 5: Subscribe

The fifth experiment proved the advantages of using IOTA’s index feature for retrieving transactions from the Tangle. According to the results shown in Figure 8b, it took P-IOTA less than 4 s to collect 100 transactions, representing alerts. The close-to-zero variance and standard deviation indicated high consistency in the time taken to collect transactions. As anticipated, Figure 8b also demonstrates that the average latency for reading transactions was significantly lower, by one order of magnitude, compared to the latency for publishing.

### 5.8. Experiment 6: Packet Filter Installation

The sixth experiment assessed the time to install a mitigation rule delivered through the IOTA layer. The rule was deployed by P-IOTA using the P4Runtime API and the Southbound Interface of P4 Section 4.2.3. Similar to the third experiment, we compared our solution to [24]. We demonstrated that P-IOTA outperformed the compared approach since we only installed one rule to perform the mitigation (Table 2). Figure 8a shows the mean, variance, and standard deviation of Experiment 6, based on 100 measurements.

### 5.9. Time and Computational Analysis

**Figure 9:**
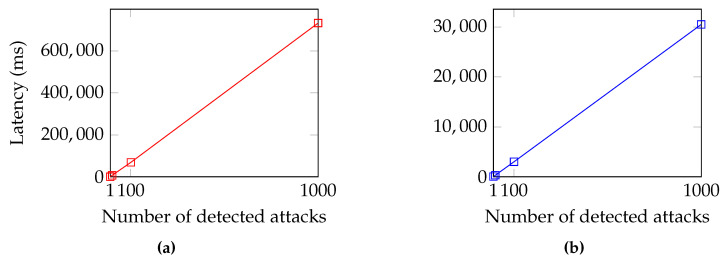
The latency trend of published detected attacks (**a**) and their retrieval (**b**).

Time and computational analysis is critical in evaluating whether our proposal can be deployed in real-world scenarios. Figure 9 outlines that, in the IOTA network, the latency increased with an approximate O(n) complexity as the number of notified attacks scaled up, both for publishing detected attacks (Figure 9a) and retrieving them (Figure 9b).

Moreover, computational considerations are essential in evaluating the practicality and efficiency of the IOTA network. The IOTA Tangle is designed to be lightweight and energy-efficient, making it ideal for deployment on low-power devices. Official experiments [35] have shown that the IOTA network can operate successfully on devices such as the Raspberry Pi 3 and 4 with very low energy consumption, ranging from 2 J to 6 J approximately. This is a significant advantage for the IOTA network, as it not only reduces its environmental impact, but also makes it more accessible and cost-effective for a wide range of applications, including those based on SDN.

**Figure 10:**
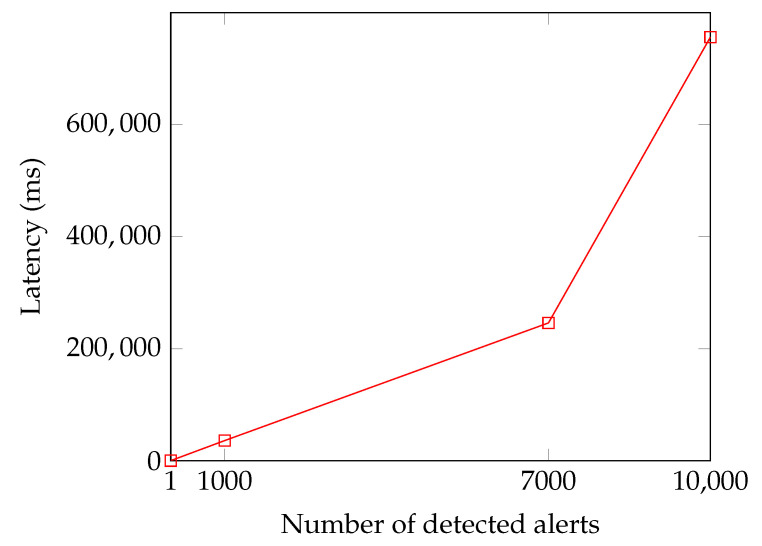
The relationship between the number of detected alerts and the latency in the Southbound Interface channel.

Regarding the SDN layer, Figure 10 illustrates the correlation between the number of alerts detected and the time required to forward them to the IOTA node. The graph shows a linear relationship for a rate of up to 7000 detected attacks. However, beyond that threshold, the latency increases gradually due to the limited bandwidth of the Southbound Interface, which has a maximum capacity of 14 Mbps in bmv2. It is worth noting that this test is not applicable to the retrieval phase. Installing thousands of rules on a switch can cause congestion in the match–action table, which should be minimally used.

### 5.10. Discussion

As a yardstick for comparison, we considered a proposal that uses Ethereum, which is one of the most-widely used blockchains. In 2022, Ethereum switched to a PoS consensus protocol, with a block-adding time of 12 s as stated in the official documentation [36]. However, adding a block to the chain does not guarantee its validity. To ensure the block’s validity, it is necessary to wait until it is finalized, meaning it cannot be modified without a significant amount of ETH getting burned. In Ethereum, this is performed through “checkpoint blocks” that are issued every 32 blocks added. If a pair of checkpoints attracts votes, representing at least 2/3 of the validators, all blocks prior to the least recent checkpoint are considered finalized. Therefore, it is necessary to wait for at least 64 blocks, approximately 12 min, to ensure block’s validity.

According to the literature [2,3,4], IOTA emerged as the best solution for the proposed case study because of its low latency, high throughput, and low power consumption. These are key features in scenarios where fast response times are necessary to mitigate attacks. Additionally, routing devices often have limited resources, making it imperative to use lightweight protocols like that of IOTA.

These considerations are also supported by the results in Table 2, which compare P-IOTA and [24] in terms of average latency. The experimental results showed that our solution significantly outperformed solutions that adopt Ethereum technologies, decreasing the time taken to alert the other nodes, including the time to forward the alert from the data to the control plane and the time to notify other nodes.

## 6. Conclusions

In this paper, we presented P-IOTA, an architecture for detecting attacks and alerting potentially affected nodes that are geographically distributed. Our proposal leverages the P4 programmable data plane to implement the detection logic and uses IOTA to disseminate alarms to nodes belonging to the same organization or, in the case of the federation, to different organizations. P-IOTA also enables keeping the history of the detected attacks.

We implemented a prototype of our solution to evaluate its performance while reporting and notifying threat about alerts during an SYN flooding attack. Specifically, we measured the latency in sending a notification and updating incorrect alerts. The experimental results demonstrated that IOTA enables these operations with a latency lower than 1 ms, outperforming traditional blockchains, which typically take minutes to confirm a block.

In light of the foregoing results, we believe that this work proves that IOTA is a promising technology for alerting nodes about threats in SDN-based environments. It can be also leveraged to handle various attack scenarios in which multiple entities need to be notified (i.e., threat intelligence). In future research, we plan to encompass mitigation policies.

## Figures and Tables

**Table 1:** Related works list of analyzed topics.

Ref.	SDN	P4	IoT	Ethereum	Recording Events	File Transfers	Security
[16]	X		X	X		X	
[17]	X				X		
[19]	X			X			X
[20]	X		X		X		X
[21]	X		X	X			X
[22]	X		X				X
[24]	X	X	X	X			X
[25]	X	X					X

**Table 2:** Comparison between P-IOTA and Febro et al. [24].

	Experiment 3	Experiment 4	Experiment 5	Experiment 6
Febro et al. [24]	64,000 ms	517,330 ms	100,000 ms	80,000 ms
P-IOTA	35.54 ms	71,640 ms	3720 ms	126.83 ms

## Data Availability

Not applicable.
